# Enhancement of the Seed-Target Recognition Step in RNA Silencing by a PIWI/MID Domain Protein

**DOI:** 10.1016/j.molcel.2008.12.012

**Published:** 2009-01-30

**Authors:** James S. Parker, Eneida A. Parizotto, Muhan Wang, S. Mark Roe, David Barford

**Affiliations:** 1 Section of Structural Biology, Institute of Cancer Research, Chester Beatty Laboratories, 237 Fulham Road, London SW3 6JB, UK; 2 Laboratory of Molecular Biophysics, Department of Biochemistry, University of Oxford, South Parks Road, Oxford OX1 3QU, UK

**Keywords:** RNA, PROTEINS

## Abstract

Target recognition in RNA silencing is governed by the “seed sequence” of a guide RNA strand associated with the PIWI/MID domain of an Argonaute protein in RISC. Using a reconstituted in vitro target recognition system, we show that a model PIWI/MID domain protein confers position-dependent tightening and loosening of guide-strand-target interactions. Over the seed sequence, the interaction affinity is enhanced up to ˜300-fold. Enhancement is achieved through a reduced entropy penalty for the interaction. In contrast, interactions 3′ of the seed are inhibited. We quantified mismatched target recognition inside and outside the seed, revealing amplified discrimination at the third position in the seed mediated by the PIWI/MID domain. Thus, association of the guide strand with the PIWI/MID domain generates an enhanced affinity anchor site over the seed that can promote fidelity in target recognition and stabilize and guide the assembly of the active silencing complex.

## Introduction

RNA silencing refers to a group of related gene silencing mechanisms mediated by short (18–30 nucleotide) single-stranded RNA molecules. These mechanisms include RNA interference (RNAi) mediated by small interfering RNAs (siRNAs), posttranscriptional gene regulation by microRNAs (miRNAs), and the suppression of parasitic mobile genetic elements by repeat-associated small interfering RNAs (rasiRNAs) and (some) Piwi-interacting RNAs (piRNAs) (Aravin et al., 2007; Filipowicz et al., 2008; Kim and Rossi, 2007; Klattenhoff and Theurkauf, 2008; Rana, 2007; Stefani and Slack, 2008). The mechanisms share a common targeting component, the single-stranded RNA known as the “guide strand,” and a common scaffolding and catalytic protein component, a member of the Argonaute protein family (Faehnle and Joshua-Tor, 2007; Hutvagner and Simard, 2008; Parker and Barford, 2006; Peters and Meister, 2007).

Target selection in RNA silencing is governed by the “seed sequence” at the 5′ end of the guide strand (Lewis et al., 2003; and references below), with important prerequisites for recognition including guide-strand and target-sequence coexpression (Farh et al., 2005; Stark et al., 2005) and accessibility of the target site (Ameres et al., 2007; Brown et al., 2005; Didiano and Hobert, 2006; Kedde et al., 2007; Kertesz et al., 2007; Long et al., 2007; Vella et al., 2004). The core of the seed sequence resides between nucleotides 2–7 of the guide strand (as measured from the 5′ end) and, to a lesser extent, can include nucleotide 8 (Lewis et al., 2005). The key role of the seed sequence is highlighted by the wealth of data for the sequences of animal microRNAs and their targets, showing this to be the outstanding region of complementarity (Baek et al., 2008; Lai, 2002; Lewis et al., 2003; Lim et al., 2005; Selbach et al., 2008; Stark et al., 2003; Wightman et al., 1993). In experimental RNAi, the identities of off-targets are determined by complementarity to the seed regions of the introduced siRNAs (Birmingham et al., 2006; Jackson et al., 2006). These empirical data are supported by biochemical studies showing the importance of seed-target complementarity for effective RNA silencing (Brennecke et al., 2005; Doench and Sharp, 2004; Mallory et al., 2004).

Paradoxically, some of the first characterized microRNA-target interactions contain imperfect seed base pairing (Lee et al., 1993; Reinhart et al., 2000; Wightman et al., 1993). Two recent studies have revealed synergistic interactions between closely spaced target sites (Grimson et al., 2007; Saetrom et al., 2007), which may account for the potency of some apparently weaker target sites, such as the *Caenorhabditis elegans lin4-lin14* interactions (Lee et al., 1993; Wightman et al., 1993). In addition, extensive 3′ base complementarity can, in some instances, compensate for weaker seed regions (Brennecke et al., 2005; Doench and Sharp, 2004), and it is proposed that these sites may constitute a distinct (though minor) class of target sites (Brennecke et al., 2005). Modulation of weaker seed match interactions by 3′ base pairing could in principle facilitate differential regulation of targets by members of a microRNA family (Brennecke et al., 2005; Lewis et al., 2005).

Seed-target recognition does not occur in isolation but in the context of large multicomponent effector complexes, such as RISC or RITS. Crystal structures show that the seed region of the guide strand is anchored via its phosphodiester backbone to the PIWI/MID domain of Argonaute and that this complex provides the platform for target mRNA recognition (Ma et al., 2005; Parker et al., 2005; Wang et al., 2008). It is believed that guide-target recognition nucleates over the seed region, before propagating toward the 3′ end of the guide (Ameres et al., 2007; Parker and Barford, 2006; Wang et al., 2008; Yuan et al., 2005). Using an atomically defined model system, we set out to recapitulate this minimal seed-target recognition step and to determine the influence of the protein on target recognition. We show, using isothermal titration calorimetry (ITC), that association with a PIWI/MID domain protein (AfPiwi from *Archaeoglobus fulgidus*) greatly enhances the affinity of the seed-target interaction. Enhancement is achieved through a reduced entropy penalty for the interaction, probably as a result of immobilization or preordering of the guide. We examined the position specificity of binding modulation by AfPiwi, showing that enhancement is strictly localized to the seed region. Guide-target interactions more 3′ along the guide, including around the scissile phosphate region of the duplex, are inhibited by AfPiwi. We also assess the capacity of the complex for mismatched target recognition, showing that mismatches can have a major influence on the affinity of seed-target recognition, with the impact of mismatches at position 3 amplified through association with the protein. We discuss potential molecular explanations for our observations and explore their implications with respect to the mechanisms of RNA silencing.

## Results

### Structure of an AfPiwi-Guide DNA-Target DNA Complex

**Figure 1:**
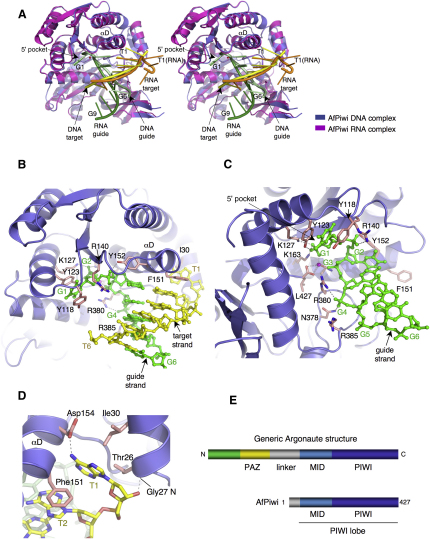
Structure of AfPiwi in Complex with Duplex DNA (A) A stereoview showing superimposition of AfPiwi in complex with duplex DNA (yellow and light green) and duplex RNA (orange and dark green) (Parker et al., 2005). (B and C) Two views showing detail of interactions between AfPiwi and duplex DNA. The target strand is omitted in (C) for clarity. (D) Detail of the T1 binding pocket. The figures were prepared using PYMOL (http://www.pymol.org/). (E) Schematic of domain organization of a generic Argonaute protein and AfPiwi. The latter is composed of the PIWI/MID domain or “PIWI lobe” of Argonaute.

Some of our experiments were performed using DNA mimics of the guide and target strands, so we first determined the structure of AfPiwi in complex with DNA mimics of guide and target strands, and compared the structure to a similar complex with RNA (Figure 1). AfPiwi is a model PIWI/MID domain protein, corresponding to the “PIWI lobe” of a full-length Argonaute protein (Parker et al., 2004; Song et al., 2004; Yuan et al., 2005) (Figure 1). The structure was determined by molecular replacement to a resolution of 1.9 Å (Table 1). Nomenclature for guide and target nucleotide positions follows that of Lewis et al. (2005) and Parker et al. (2005), with G and T designating guide and target, respectively, and numbering beginning from the base pair at the 5′ end of the guide (Figure 1).

The structure superimposes closely with the RNA complex (Parker et al., 2005; Ma et al., 2005) (Figure 1A), with the guide strand anchored to AfPiwi via its 5′ phosphate group (Figures 1B and 1C) and the phosphodiester backbone of the seed region (Figure 1C). At the 5′ end, the nucleic acid duplex is distorted such that the G1 and T1 bases are unwound with G1 being displaced into the conserved 5′ binding pocket (Figure 1A). The target strand interacts primarily with the base edges of the guide. The principal differences are the formation of B-form duplex rather than A-form duplex (see Figure S1 available online) and the insertion of the DNA T1 target nucleotide into a pocket in AfPiwi, forming interactions with Ile30, Phe151, and Asp154 (Figure 1D). In contrast, in the RNA complex, the T1 nucleotide is significantly distorted and exposed entirely to solvent (Figure 1A), indicating that the RNA nucleotide is incompatible with the binding pocket. The interactions between conserved residues in AfPiwi and the phosphodiester backbone of the seed region of the guide are identical or similar in most cases to those in the RNA complex (Figures 1B and 1C and Supplemental Data).

Prokaryotic Argonaute proteins display greater slicing activity using DNA guide strands (Yuan et al., 2005), consistent with the apparent evolutionary relationship with RNase H (Parker et al., 2004; Song et al., 2004). AfPiwi binds DNA more tightly than RNA (Ma et al., 2005). The higher affinity for DNA facilitated complex formation and analysis by ITC in some of our experiments (below).

### Enhancement of the Seed-Target Interaction

**Figure 2:**
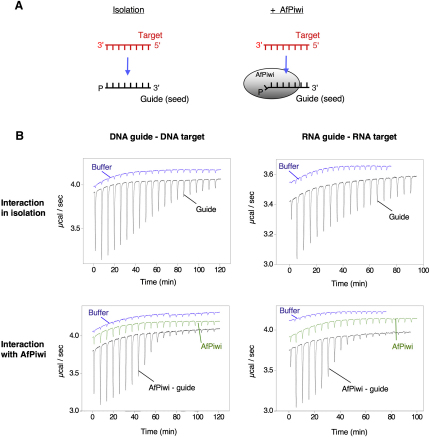
Overview of Experimental Strategy (A) Schematic representation of the binding experiments. (B) Typical raw ITC data for DNA (left) and RNA (right) seed-target interaction experiments in isolation (top) and in the presence of AfPiwi (bottom). Target-guide heats (±AfPiwi) are shown in black, target-buffer heats in blue, and target-AfPiwi heats in green. The data provided the binding isotherms shown in Figures 3A and 3B.

The basis for our experiments and examples of the raw data obtained by ITC are shown in Figures 2A and 2B, respectively. ITC provides an accurate measure of the affinity (K_d_), enthalpy (ΔH), and entropy (ΔS) of an interaction by measuring the heat released or absorbed when a ligand is titrated into a solution of a macromolecule (Pierce et al., 1999). We use ITC to compare these thermodynamic parameters for the interaction of a guide strand and a target strand in isolation (Figure 2A, left, and Figure 2B, top) and when the guide strand forms a complex with AfPiwi (Figure 2A, right, and Figure 2B, bottom). Thus, we are able to determine the influence of association of the guide strand with the PIWI/MID domain, a complex that more truly represents the target recognition platform in the mechanisms of RNA silencing (Parker et al., 2005; Ma et al., 2005; Wang et al., 2008). In the experiments, the guide strands are 5′ phosphorylated to interact with the conserved 5′ binding pocket in AfPiwi, whereas the target strands are unphosphorylated. The lengths of the annealing regions were selected to yield the most accurate data by ITC (i.e., with affinities in the μM to nM range, under the conditions we use). Argonaute proteins have the capacity to interact with guide and target strands of almost any sequence (reflected in the absence of base specific contacts in determined structures) so sequences were chosen merely to avoid self-self or ambiguous guide-target interactions.

**Figure 3:**
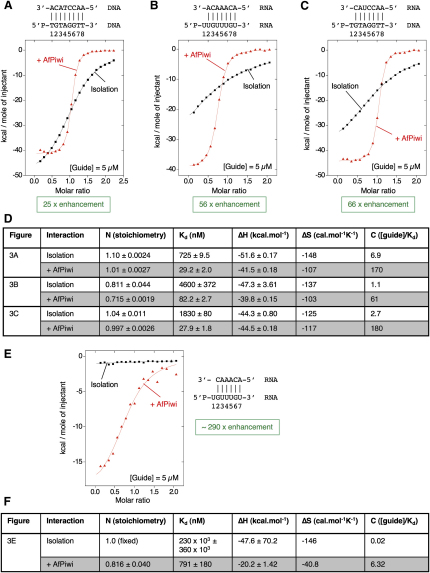
Enhancement of the Seed-Target Interaction (A–C) ITC binding isotherms for DNA and RNA guide (seed)-target interactions in isolation (black, squares) and in the presence of AfPiwi (red, triangles). Annealed duplex structures (guide at the bottom, target at the top) are shown over the corresponding charts; nucleotides are numbered from the 5′ ends of the guide strands. Raw data for (A) and (B) are shown in Figure 2B and (C) in Figure S2. (D) Derived thermodynamic parameters for the interactions shown in (A)–(C). (E) Rescue of a “melted” seed-target interaction by AfPiwi. ITC binding isotherms for the interactions in isolation (black, squares) and in the presence of AfPiwi (red, triangles) and the annealed duplex structure (guide at the bottom, target at the top) are shown. Raw data are shown in Figure S2. (F) Derived thermodynamic parameters for the interactions shown in (E).

Figures 3A–3C show the influence of AfPiwi on the annealing of oligonucleotides mimicking the guide seed (nucleotides 2–7/8) − target interaction. We studied the interactions of a DNA guide and DNA target (Figure 3A), RNA guide and RNA target (Figure 3B), and DNA guide and RNA target (Figure 3C). In all instances, we observe a significant increase in the affinity of the interaction in the presence of AfPiwi, represented as an increase in steepness of the (exothermic) ITC titration curves (Experimental Procedures). For DNA/DNA, we observe a 25-fold increase in affinity (725 nM to 29 nM), for RNA/RNA a 56-fold increase in affinity (4.6 μM to 82 nM), and for DNA/RNA a 66-fold increase in affinity (1.83 μM to 28 nM). The heats obtained from titration of the target strand into AfPiwi alone are small (Figure 2B, bottom), so would not be responsible for modulation of the binding curve. As a further control, ITC analysis shows that duplex interaction with AfPiwi is an endothermic interaction (Figure S3), so the curves do not reflect the interaction of newly formed duplex with AfPiwi. In addition to the ITC data, binding studies using gel filtration are consistent with an enhanced interaction in the presence of AfPiwi (Figure S4). Together, the results show that association of the seed region of a guide strand with a PIWI/MID domain protein results in a greatly enhanced affinity for a corresponding target strand.

Thermodynamic parameters for the interactions are displayed in Figure 3D. In general, nucleic acid strand association is an exothermic reaction driven by enthalpy and counteracted by an unfavorable loss of entropy (an entropy penalty). The data in Figure 3D show that the interactions are tighter in the presence of AfPiwi because the losses in entropy upon strand association are lower. (The favorable exothermic enthalpies are equal or lower in the presence of AfPiwi.) This could be explained by the preordering and/or immobilization of the guide strands by AfPiwi. In other words, through the influence of AfPiwi on the entropy of the guide strand, some of the guide strand binding energy to AfPiwi can be transferred to the guide-target-strand interaction. There is no structure of AfPiwi in complex with single stranded nucleic acid to assess the degree of preordering of the guide. However, the recent crystal structure of *Thermus thermophilus* full-length Argonaute bound to a 21-mer DNA guide strand (no target strand) (Wang et al., 2008) shows that nucleotides 2–11 of the guide are pre-ordered by the protein, whereas the nucleotides 12–18 are completely disordered, despite tethering at both ends. Our data would suggest that preordering at the 5′ end of the guide is maintained to promote the seed-target interaction.

Figure 3E shows the enhancement of an RNA hexamer guide-target interaction, representing the core of a seed sequence (nucleotides 2–7). Significantly, this interaction is almost undetectable in isolation. The predicted melting temperature for the interaction is 7.5°C (DINAMelt server), substantially below the temperature used in the ITC experiments (20°C). (By comparison, the interactions shown in Figures 3A and 3B have predicted melting temperatures of 26.5°C and 22.7°C, respectively.) Therefore, in addition to the enhancement of preexisting interactions, AfPiwi is capable of rescuing interactions that would otherwise be destabilized due to temperature. A best estimate of the affinity of this interaction in isolation is ˜230 μM (Figure 3F), implying, in this case, an enhancement of nearly 300-fold. Stabilization of otherwise melted seed-target nucleation interactions, particularly at the higher mammalian cell temperature of 37°C, could be an important aspect of PIWI/MID domain function.

The RNA/RNA and DNA/RNA enhancement experiments presented in this section were conducted using target strands lacking a T1 nucleotide to maintain the same number of base pairs in isolation and in the presence of AfPiwi (the G1-T1 base pair is unwound in the presence of AfPiwi). Measurement of the enhancement of a similar RNA-RNA interaction with a flush 5′ end results in an apparent reduced enhancement (enhancement of ˜5-fold, Figure S5), as the loss of the G1-T1 base pair depletes the enhancement obtained over the seed region. By contrast, the DNA/DNA strand interaction displays a similar level of measured enhancement with a flush 5′ end (25-fold, Figure 3A) and in the absence of a T1 nucleotide (21-fold, Figure S6), indicating that the insertion of the T1 nucleotide into the T1 binding pocket in AfPiwi compensates for the loss of the base pair. Furthermore, the crystal structures of the AfPiwi RNA/RNA and DNA/DNA complexes show that in both cases target nucleotides T2 to T8 make no contacts to the protein, interacting with the complex through base pairing to the guide only. Thus, consistent with the thermodynamic analysis, the structures show that enhancement is achieved without any additional direct contacts to the target strand and purely through the influence of AfPiwi on the guide.

### Seed-Specific Enhancement and 3′ Inhibition

**Figure 4:**
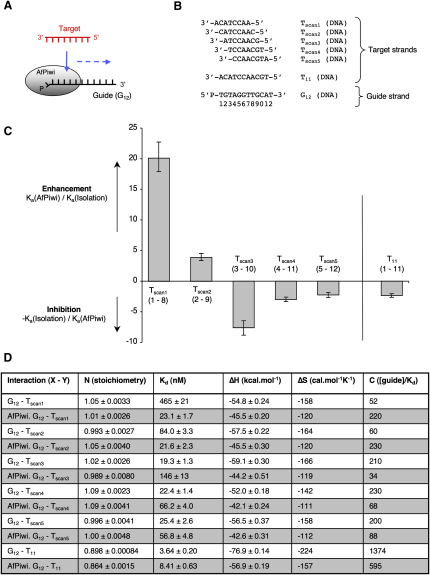
Scanning Along the Guide Strand (A) Schematic representation of the scanning experiments. (B) Strand sequences. (C) Fold-binding enhancement or inhibition mediated by AfPiwi. Error bars are calculated as the combined errors for the affinities in isolation and in the presence of AfPiwi derived from the errors during curve fitting. (D) Derived thermodynamic parameters for the interactions described in (A)–(C). Binding isotherms and raw titration data are shown in Figures S7–S9.

We wanted to assess whether there was any variation in the influence of AfPiwi at different positions along the guide strand. We therefore performed a series of “scanning” experiments using a 12-mer DNA guide (G_12_) and a series of step-translated 8-mer DNA target strands (T_scan1_ to T_scan5_) (Figure 4). The G_12_-T_scan1_ interaction, covering nucleotides 1–8 of the guide, was enhanced 20-fold by AfPiwi, similar to the interaction shown in Figure 3A. The G_12_-T_scan2_ interaction, covering nucleotides 2–9, was enhanced to a lesser extent (3.9-fold). Because a DNA-DNA interaction covering nucleotides 2–8 is enhanced 21-fold (previous section, Figure S6), this suggests AfPiwi imparts a degree of inhibition at position 9 of the guide. Strikingly, the interactions covering nucleotides 3–10 (T_scan3_), 4–11 (T_scan4_), and 5–12 (T_scan5_) are all inhibited, to a small extent (between 2.2-fold and 7.6-fold), by AfPiwi. Thus, toward the 3′ end of the guide, beginning at around position 9, AfPiwi weakens the guide-target interaction.

To assess the net effect of enhancement over the seed region and inhibition at the 3′ end, we determined the influence of AfPiwi on the interaction of an 11-mer DNA target (T_11_) with G_12_, covering nucleotides 1–11 of the guide (Figure 4). Despite a degree of inaccuracy in the determination of the affinity of this interaction in isolation (owing to its strength, the interaction lies outside the dynamic range of the ITC with a C value of > 1374 [see Experimental Procedures]), the data indicate that the interaction is weaker in the presence of AfPiwi (see Figure S9). A best-fit analysis yields an inhibition of 2.3-fold, from 3.6 nM to 8.4 nM (Figure 4D). The result has implications for understanding the regeneration of RISC following passenger or target strand cleavage, demonstrating how, despite an enhanced affinity over the seed region, the 3′ fragment of the passenger or target strand can display an overall diminished affinity for the complex. Note that longer guide-target interactions (e.g., 21 nucleotide) possess affinities that lie far outside the dynamic range of ITC and cannot be measured with any accuracy. Given also that AfPiwi does not possess a PAZ domain, which interacts with the 3′ terminus of a full-length (21 nucleotide) guide (Wang et al., 2008), our analysis has focused primarily on effects within the 5′ portion of the guide.

### Mismatched Target Interactions

Complementarity over the seed region is particularly important in target strand selection. This is evident from studies of microRNAs and their targets (Baek et al., 2008; Selbach et al., 2008) and also from experimental manipulation of RNA silencing systems (Brennecke et al., 2005; Doench and Sharp, 2004; Mallory et al., 2004). To understand the requirement for complementarity over the seed region, we wished to assess the impact of mismatches on our reconstituted seed-target nucleation event, in light of the observed binding modulation over the seed region. Mismatches, and the lengths of the guide and target strands used, were chosen to yield affinities within the optimal dynamic range of the ITC (as far as possible), to provide maximal accuracy and affinity resolution.

**Figure 5:**
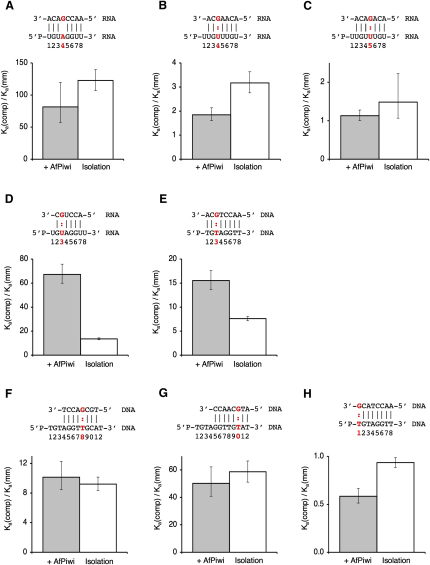
Mismatched Target Recognition (A–H) Impact of mismatches on guide-target recognition in the presence of AfPiwi (gray bars) and in isolation (white bars). The bars display the difference in affinity between the complementary (comp) and mismatched (mm) interactions. Error bars are calculated as the combined errors for the complementary and mismatched affinities derived from the errors during curve fitting. The annealed structures of the corresponding mismatched interactions (guide, lower and target, upper, with mismatches highlighted in red) are shown over the charts. Derived thermodynamic parameters for the interactions are shown in Table 2. Binding isotherms and raw titration data are shown in Figures S10–S12.

A single A:G mismatch at position 4 in the seed region resulted in an ˜82-fold loss in affinity for the RNA seed-target interaction in the presence of AfPiwi, compared with the complementary interaction (Figure 5A, gray bar). This is a substantial drop that would appear to be consistent with target deselection. The A:G mismatch is one of the least severe nonwobble mismatches, suggesting other mismatches would result in greater reductions. We also compared the complementary and A:G mismatched interactions in the absence of AfPiwi (Figure 5A, white bar). Notably, we obtain a comparable drop in affinity (although the absolute values are different; Table 2), indicating that association with the protein platform and the binding modulatory activity do not significantly alter the magnitude of the influence of the mismatch. (We note that the affinity for this particular seed-target interaction is slightly weaker in the presence of AfPiwi [Table 2], which very likely reflects the negative impact of the loss of the G1-T1 base pair, which was included to bring the affinities of the interactions within the dynamic range of the ITC.)

The G:U wobble mismatch in RNA adopts favorable base pairing and has a stability comparable (though slightly lower) to that of Watson-Crick base pairs (Freier et al., 1986; Varani and McClain, 2000). Some reports suggest that G:U wobble base pairs are detrimental within the seed region (Brennecke et al., 2005; Doench and Sharp, 2004), whereas others show that in many instances they are tolerated (Didiano and Hobert, 2006). To assess the impact of G:U wobble mismatches on the seed-target nucleation event, we have introduced G:U wobble base pairs at positions 3, 4, and 5 along the guide strand and determined their influence on recognition.

G:U wobble mismatches at positions 4 and 5 have a relatively mild impact on seed-target recognition in the presence of AfPiwi (˜1.8-fold and ˜1.1-fold, respectively; Figures 5B and 5C, gray bars). This is consistent with the favorable nature of their interactions. By contrast, the G:U wobble mismatch at position 3 resulted in a ˜67-fold loss in affinity (Figure 5D, gray bar). Thus, the results reveal a striking variation in the impact of G:U wobbles on seed-target recognition. Comparison of the data with the equivalent interactions in the absence of AfPiwi (Figures 5B–5D, white bars) shows that this variation is, in most part, due to association of the seed with AfPiwi. Although the detrimental impact of G:U wobbles base pairs at positions 4 and 5 is very similar in the presence and absence of AfPiwi (Figures 5B and 5C), the impact of the G:U wobble at position 3 is very much milder in isolation (only ˜14-fold) than in the presence of AfPiwi (Figure 5D, compare gray and white bars). Thus, association of the seed region of the guide with AfPiwi amplifies the discrimination for the G:U wobble at position 3. This is achieved through enhancement of the complementary interaction by AfPiwi and inhibition of the mismatched interaction (Table 2). The result is supported by a similar effect demonstrated for DNA strands of different sequence with a T:G mismatch at position 3 (Figure 5E). The sensitivity to mismatches at position 3 conferred by AfPiwi may be related to the numerous structural contacts mediated by the phosphate group of the guide strand at position 3 (see Discussion).

Mismatches outside the seed region are common in animal microRNAs and appear to be better tolerated than mismatches within the seed. We tested the localized impact of T:G mismatches on guide-target interactions at the border of the seed region (position 8; Figure 5F) and just outside the seed (position 10; Figure 5G), using the scanning 3′ target strands (DNA) discussed in the previous section. The mismatches result in moderate losses in guide-target affinity in the presence of AfPiwi over the annealing regions (10-fold loss at position 8 and 50-fold at position 10; gray bars). Notably, comparison with the interactions in isolation (Figures 5F and 5G, white bars) shows that the impact of the mismatches is not influenced by AfPiwi. Therefore, the data are inconsistent with a mechanism whereby the protein diminishes the impact of mismatches outside the seed (which could explain their frequency) suggesting instead they are tolerated because they are part of a secondary recognition event (duplex propagation) after formation of the strong seed anchor.

We also tested a mismatch at position 1 in DNA guide-target recognition (Figure 5H), in light of the unwinding of the G1-T1 base pair in the presence of AfPiwi and the insertion of the T1 nucleotide into a pocket in AfPiwi. Strikingly, the target strand mismatched to the guide strand at position 1 (T:G mismatch) showed tighter binding than the complementary strand (1.7-fold), consistent with the absence of G1-T1 recognition in this complex and perhaps illustrating a base preference for the T1 binding pocket. It has previously been observed that under certain conditions a G1-T1 mismatch can enhance the activity of RISC (Haley and Zamore, 2004). The data may also be relevant to the observed preference for adenine at the T1 position in microRNA targets (Lewis et al., 2005).

## Discussion

### Enhancement of Nucleic Acid Strand Interactions through Entropy Modulation

We show that association of the 5′ seed region of a guide RNA or DNA strand with a model PIWI/MID domain protein, a complex that mimics the target recognition platform of RNA silencing mechanisms, results in a greatly enhanced affinity for a corresponding target strand. The enhancement is greatest with a guide DNA strand and a target RNA strand, which is consistent with reported strand preferences for other prokaryotic Argonaute proteins (Wang et al., 2008; Yuan et al., 2005) and reflects their evolutionary relationship with RNase H family enzymes (Parker et al., 2004; Song et al., 2004). However, despite this insight, the functions of the prokaryotic Argonaute proteins, which currently serve as models for understanding the molecular mechanisms of eukaryotic Argonaute proteins (Wang et al., 2008), remain unclear.

Insight into the mechanism of enhanced affinity is obtained from the thermodynamic analyses of the binding reactions provided by ITC. In all instances, the data show that binding is tighter in the presence of AfPiwi because the entropy penalty incurred when the strands associate is smaller. This is very likely to reflect the preimmobilization or preordering of the isolated guide strand by AfPiwi, thereby diminishing the overall loss in strand mobility upon duplex formation. By this mechanism, AfPiwi can transfer binding energy from association with the guide strand to formation of the guide (seed)-target duplex. Consistent with these predictions, the recent crystal structure of *Thermus thermophilus* Argonaute bound to an isolated guide (DNA) strand (Wang et al., 2008) shows that the 5′ seed-containing region of the guide strand is indeed ordered by the protein, whereas the majority of the 3′ portion of the guide is disordered and mobile, despite being tethered at both ends. The importance of the influence of the protein on the conformation of the guide strand is reinforced by our observation, from crystallography, that enhancement is achieved without any contacts from the protein to the target strand.

These observations are reminiscent of the properties of Locked Nucleic Acids (LNAs), which also confer enhanced affinity for complementary nucleic acid strands (McTigue et al., 2004; Vester and Wengel, 2004). LNA nucleotides have a methylene group linking the 2′ and 4′ carbons on the ribose ring, reducing conformational mobility and favoring a 3′-*endo* conformation found in A-form duplexes. The enhanced binding affinity is in part attributed to the influence of preorganization, thereby modulating the entropy of the interactions. LNA-containing duplexes also display substantially increased melting temperatures. In general, the mobility of single stranded nucleic acids and the increase in order concomitant with their interaction may render them particularly susceptible to binding enhancement by this mechanism. It seems likely that nucleic acid interactions in other macromolecular complexes will be subject to similar effects.

### Implications for the Mechanism of RISC

The Argonaute-guide-strand complex forms the core of the RNA silencing effector complexes RISC and RITS (Hammond et al., 2001; Verdel et al., 2004). It is known that Argonaute provides the framework for the interactions of the guide and target strands and the various other components of the complexes, such as Dicer and TRBP (in RISC) and Chp1 and Tas3 (in RITS). However, the molecular mechanisms operating within RISC are incompletely understood, and it is unclear how the multiple activities of RISC (or RITS)—target strand recognition, active complex assembly, the choice between slicing and retention, and complex recycling—are accomplished. Here, we show that a protein corresponding to the PIWI/MID domain of Argonaute, which together with the guide strand forms the target recognition platform of RISC or RITS, is capable of variously modulating the affinity of guide-target interactions. Over the seed region, which constitutes the primary recognition site for a target, the guide-target interaction is greatly enhanced. In contrast, 3′ of the seed region (beginning at around nucleotide 9 of the guide and covering the scissile phosphate region), the guide-target interaction is loosened. Thus, the protein component associated with the guide-target duplex is capable of a variable position-dependent influence on guide-target interactions.

Association of the seed region of the guide strand with the PIWI/MID domain protein generates a high-affinity recognition site for the target, with an affinity far exceeding that for the corresponding interaction in isolation. Furthermore, this extends to rescue of otherwise “melted” seed-target interactions, whose interaction would otherwise be completely abolished as the ambient temperature exceeds the melting temperature of the duplex. The short length of the seed region (six or seven nucleotides) renders this region particularly susceptible to this effect, which would be even more pronounced in the higher temperature environment of mammalian cells.

In addition, the strong seed anchor site may stabilize and facilitate the subsequent steps in the formation of the active slicing complex, where it appears the guide-target duplex must be propagated through Argonaute accompanied by extensive conformational change (Yuan et al., 2005). Because the apparent overall affinity of the target strand for the RISC complex is massively lower than the predicted affinity for a ˜20-mer guide-target duplex (Ameres et al., 2007; Haley and Zamore, 2004) it is clear that formation of the functional complex is accompanied by extensive conformational strain. The formation of a strong nucleation anchor may be a prerequisite for the subsequent steps in complex assembly. It is also conceivable that preferential interaction at the seed region could be required to drive productive complex assembly, if the conformational changes in the complex are dependent on nucleation here followed by 5′ to 3′ duplex propagation.

Perhaps unexpectedly, guide-target interactions 3′ of the seed region are weakened by AfPiwi. This asymmetry in activity (enhancement over the seed and inhibition at more 3′ positions) is consistent with previous observations for the RISC complex as a whole, showing preferential target interaction at the 5′ end of the guide (Ameres et al., 2007; Haley and Zamore, 2004). The inhibitory activity of the protein may be important for the release of the passenger or target strand fragments following slicing, particularly in light of the strong enhancement directly over the seed region. More speculatively, weakening of guide-target interactions around the scissile phosphate of the target (between nucleotides 10 and 11) could reinforce the decision of whether to slice the target, amplifying the effects of mismatches at this site (as indeed we observe for the mismatch at position 10). In any case, it is clear that (1) position-dependent effects on guide-target interactions facilitate the catalytic process of RISC and (2) the stark transition in modulatory activity we observe around position 9 highlights the importance of the seed region in target recognition. We also note that, in addition to these effects, PAZ-domain mediated interactions with the 3′ end of the guide strand in full length Argonaute will have an influence on guide-target interactions in the 3′ region.

### Target Selection in RNA Silencing

Target selection in RNA silencing is determined by accessibility of a target site in a long target RNA—which can be affected by *trans*-binding proteins (Kedde et al., 2007) or *cis*-acting RNA secondary structure (Ameres et al., 2007; Brown et al., 2005; Kertesz et al., 2007; Long et al., 2007)—and complementarity to the seed region of the guide. Here we have quantified mismatched target recognition in the context of the PIWI/MID domain. A nonwobble, though relatively “mild,” A:G mismatch at position 4 in the seed region resulted in a substantial depletion in guide-target affinity (˜82-fold), an affinity change that would be consistent with deselection of the target. G:U wobble mismatches have a variable influence on the recognition event, in part determined by the sequence context of the mismatch. In two instances (positions 4 and 5), the impact of the mismatch is relatively mild (1–2-fold reduction in seed-target affinity) reflecting the favorable base pairing potential of the wobble and consistent with reports that G:U wobbles in the seed do not always abrogate guide strand function (Didiano and Hobert, 2006). By contrast, a G:U wobble mismatch at position 3 resulted in a more substantial reduction in affinity (˜67-fold), notable in particular because this exceeds the reduction in affinity in the absence of the protein (here only 14-fold). Thus, at this position, the protein amplifies the impact of the mismatch, making the target substantially less favorable. Crystal structures (here and Ma et al., 2005 and Parker et al., 2005) show that the backbone phosphate of nucleotide 3 is a nexus in the interactions between AfPiwi and the guide strand, contacting both the 5′ binding metal ion (as one of the six coordinating ligands) and, in the RNA duplex, the conserved residue Arginine-380. We suggest that this position is particularly sensitive to perturbation of the duplex structure, which would result as a consequence of the G:U wobble (Mueller et al., 1999; Varani and McClain, 2000). A previous study has demonstrated a disproportionate negative influence for seed G:U wobbles in an RNA silencing system (breaking an observed correlation with theoretical annealing stability), with a significant impact for a wobble at position 3 (Doench and Sharp, 2004).

In summary, our data are consistent with a picture whereby the seed region is the primary recognition site, whose relatively short length renders it sensitive to single mismatches. Mismatches outside the seed region—which may be advantageous in RISC in order to prevent slicing (for microRNAs) and facilitate complex recycling—are tolerated as part of a secondary recognition region, dependent on the establishment of a strong recognition anchor over the seed.

## Experimental Procedures

### Protein and Oligonucleotides

AfPiwi was purified as described (Parker et al., 2004), with the inclusion of an excess (30 U/ml) of Benzonase nuclease (Novagen) in the lysis buffer to deplete nucleic acids and the incorporation of a heat shock step following cell lysis (70°C for 20 min). DNA oligonucleotides were purchased from ATDBio Ltd. and were purified by HPLC (5′ phosphorylated) or gel filtration (unphosphorylated). RNA oligonucleotides were purchased from Dharmacon Inc., purified by desalting, and converted to the 2′-hydroxyl form.

Oligonucleotide extinction coefficients were determined using the Ambion Extinction Coefficient Calculator, available at http://www.ambion.com/techlib/misc/oligo_calculator.html.

The AfPiwi extinction coefficient (82740 M^−1^cm^−1^) was determined using the Expasy ProtParam tool, available at http://us.expasy.org/tools/protparam.html.

Predicted oligonucleotide melting temperatures were determined using the DINAMelt Server (Markham and Zuker, 2005), available at http://www.bioinfo.rpi.edu/applications/hybrid/twostate.php.

### Crystallization

The sequence of the DNA (a 5′ phosphorylated self-associating 16-mer) was the same as the sequence of RNA used in a previously described structure (Parker et al., 2005) (thymine rather than uracil). Similar crystallization and cryoprotection conditions were used. Data were collected at ID14.EH2 at the ESRF.

### Structure Determination

X-ray diffraction data were processed and scaled using Mosflm (Leslie, 2006) and SCALA (CCP4, 1994), respectively. The structure was solved by molecular replacement using 2BGG (Parker et al., 2005) using PHASER (McCoy et al., 2007) and rebuilt by Arp/warp, refined using Refmac and further manual building using COOT (Emsley and Cowtan, 2004). Crystallographic data are listed in Table 1.

### ITC Analysis

Most samples were dialyzed overnight at 4°C prior to ITC analysis. The following buffer was used: 50 mM Tris (pH 7.5), 150 mM KCl, 5% (v/v) glycerol, 10 mM MgCl_2_, and 1 mM DTT. RNA and DNA oligonucleotides (of relatively small size) were dialyzed using Spectra/Por Biotech Cellulose Ester (CE) dialysis membrane (MWCO 500 Da) (Spectrum Laboratories Inc). The RNA guide strand used in Figures 5A and 5D could not be dialyzed (the binding stoichiometry becomes severely depleted, which we attribute to self-association), so, in these experiments, the RNA samples were diluted directly from concentrated stock solutions (ensuring cell and syringe buffer compositions remained identical). Samples were not degassed because this resulted in baseline “stepping.” Runs repeated with and without degassing yielded identical data.

Samples concentrations were chosen to optimize ITC C values ([macromolecule] / dissociation constant) but were limited at the lower end by the magnitudes of the heats and at the upper end by the solubility of AfPiwi. Most runs were conducted with C values in the range of 1 to 300; for an accurate determination of the binding constant, a C value between 1 and 1000 is required. (Large C values prohibit the determination of *K*_a_ because the transition is very sharp and too few titration points are collected near equivalence [Wiseman et al., 1989].) Typically, guide strand would at 3.5–24 μM in the cell and target strand at a 10-fold higher concentration in the syringe. AfPiwi would be present at a 3–4 fold excess (over the guide) in the cell to ensure full association of the guide and because trace amounts of nucleic acid readily copurify with AfPiwi depleting the “active fraction.”

Analysis was performed on a VP-ITC instrument (Microcal LLC) at 20°C. Two schedules were used: (1) dummy injection followed by 16 × 17 μl injections (2 s/μl), and (2) dummy injection followed by 19 × 15 μl injections (2 s/μl). Injection spacing was usually 360 s (to obtain flat baseline between injections), stirring was at 300 rpm, and the reference power was either 5 or 10 μcal/s.

Data analysis was performed using Origin versions 5.0 and 7.0. These programs model experimental isotherms according to equations defined by Wiseman et al. (1989) that relate the heats released or absorbed during binding titrations to the thermodynamic parameters ΔH and association constant K_a_ (see also Pierce et al., 1999). Baseline fitting and the definition of peak boundaries were performed automatically with manual correction, without bias. Heats of dilution of target strands were determined either from target strand titration into buffer (interactions in isolation), the heat of dilution at complex saturation (tight interactions in the presence of AfPiwi with C values >30), or as a fixed value compatible with the “One Set of Sites” binding model (weaker interactions in the presence of AfPiwi with C values <30) and subtracted from experimental titrations. All curves were fitted as 1:1 interactions using the Origin nonlinear least-squares fitter (“One Set of Sites” model). As can be seen from visual inspection of the curves, the majority of runs, even those involving complexes, display close proximity to ideal behavior. Affinities are quoted as dissociation constants with the errors from the Origin-calculated association constants transferred as the same fractions of primary values. All raw data and fitted binding isotherms are displayed in the main figures or Supplemental Data.

## Figures and Tables

**Table 1:** Crystallographic and Parameter Statistics

Data Set (Highest Resolution Shell in Parentheses)	
AfPiwi − DNA complex	
a (Å)	51.84
b (Å)	61.38
c (Å)	103.54
α (°)	75.80
β (°)	75.86
γ (°)	79.47
Space group	P1
Z	2
X-ray source	ESRF ID14.EH2
Wavelength (Å)	0.9330
Resolution Limit (Å)	30−1.9 (2.0−1.9)
Number of observations	187,556
Completeness (%)	95.9 (95.5)
Multiplicity	2.1 (2.1)
R_merge_	0.053 (0.42)
I/σI	11.5 (2.3)

Refinement (Highest Resolution Shell in Parentheses)	

Resolution range (Å)	30−1.9 (1.95−1.90)
Protein atoms (no.)	6370
DNA atoms (no.)	454
Ligand atoms (no.)	2 (Mn)
Solvent atoms (no.)	411
R_cryst_	0.205 (0.282)
R_free_	0.243 (0.325)
Mean B (Å^2^)	33
Rmsd bond lengths (Å)	0.019
Rmsd bond angles (°)	1.766

**Table 2:** Thermodynamic Parameters for the Interactions Shown in Figure 5

Figure	Interaction	N (stoichiometry)	K_d_ (nM)	ΔH (kcal. mol^−1^)	ΔS (cal. mol^−1^K^−1^)	C ([guide]/K_d_)
5A	isolation, complementary	0.863 ± 0.0019	6.48 ± 0.62	−58.9 ± 0.24	−164	620
	isolation, mismatch	0.874 ± 0.0075	794 ± 30	−52.1 ± 0.66	−150	5.0
	+AfPiwi, complementary	0.919 ± 0.0044	14.2 ± 2.0	−38.6 ± 0.31	−95.7	280
	+AfPiwi, mismatch	1.146 ± 0.049	1160 ± 260	−29.6 ± 2.1	−73.8	3.5
5B	isolation, complementary (as Figure 3B)	0.811 ± 0.044	4600 ± 372	−47.3 ± 3.6	−137	1.1
	isolation, mismatch	0.80 (fixed)	14600 ± 840	−46.6 ± 1.3	−137	0.82
	+AfPiwi, complementary (as Figure 3B)	0.715 ± 0.0019	82.2 ± 2.7	−39.8 ± 0.15	−103	61
	+AfPiwi, mismatch	0.691 ± 0.0080	152 ± 16	−46.4 ± 0.74	−127	33
5C	isolation, complementary (as Figure 3B)	0.811 ± 0.044	4600 ± 372	−47.3 ± 3.6	−137	1.1
	isolation, mismatch	1.02 ± 0.19	6820 ± 1900	−37.0 ± 10	−102	0.73
	+AfPiwi, complementary (as Figure 3B)	0.715 ± 0.0019	82.2 ± 2.7	−39.8 ± 0.15	−103	61
	+AfPiwi, mismatch	0.764 ± 0.0053	92.9 ± 8.0	−43.9 ± 0.43	−117	54
5D	isolation, complementary	0.938 ± 0.0015	94.7 ± 2.1	−46.8 ± 0.12	−127	53
	isolation, mismatch	0.984 ± 0.0085	1280 ± 49	−44.0 ± 0.59	−123	3.9
	+AfPiwi, complementary	0.926 ± 0.0017	30.2 ± 1.3	−33.6 ± 0.11	−80.3	170
	+AfPiwi, mismatch	1.072 ± 0.019	2030 ± 150	−40.3 ± 1.30	−111	2.5
5E	isolation, complementary	1.11 ± 0.0040	990 ± 37	−50.6 ± 0.25	−145	24
	isolation, mismatch	1.14 ± 0.0046	7520 ± 150	−46.6 ± 0.32	−135	3.2
	+AfPiwi, complementary (as Figure 3A)	1.01 ± 0.0027	29.2 ± 2.0	−41.5 ± 0.18	−107	170
	+AfPiwi, mismatch	1.16 ± 0.0086	452 ± 27	−35.2 ± 0.38	−91.2	11
5F	isolation, complementary (as Figure 4D)	1.09 ± 0.0023	22.4 ± 1.4	−52.0 ± 0.18	−142	230
	isolation, mismatch	1.057 ± 0.0044	206 ± 7.9	−47.3 ± 0.27	−131	19
	+AfPiwi, complementary (as Figure 4D)	1.09 ± 0.0041	66.2 ± 4.0	−42.1 ± 0.24	−111	68
	+AfPiwi, mismatch	0.987 ± 0.022	672 ± 82	−36.8 ± 1.24	−97.2	6.0
5G	isolation, complementary (as Figure 4D)	0.996 ± 0.0041	25.4 ± 2.6	−56.5 ± 0.37	−158	200
	isolation, mismatch	1.059 ± 0.0062	1490 ± 40	−49.5 ± 0.47	−142	3.3
	+AfPiwi, complementary (as Figure 4D)	1.00 ± 0.0048	56.8 ± 4.8	−42.6 ± 0.31	−112	88
	+AfPiwi, mismatch	0.898 ± 0.046	2850 ± 350	−44.8 ± 3.5	−127	1.8
5H	isolation, complementary	1.11 ± 0.0040	990 ± 37	−50.6 ± 0.25	−145	24
	isolation, mismatch	1.07 ± 0.0021	926 ± 16	−49.5 ± 0.13	−141	18
	+AfPiwi, complementary (as Figure 3A)	1.01 ± 0.0027	29.2 ± 2.0	−41.5 ± 0.18	−107	170
	+AfPiwi, mismatch	1.07 ± 0.0021	17.0 ± 1.1	−37.6 ± 0.13	−92.7	290

## Accession Numbers

Coordinates and structure factors have been deposited in the Protein Data Bank under accession number 2w42.

## Supplemental Data

Document S1. Supplemental Experimental Procedures, Results, Supplemental References, and 12 Figures
