# Supplementary material for: Enhancement of the Seed-Target Recognition Step in RNA Silencing by a PIWI/MID Domain Protein
Source: Mol Cell. 2009 Jan 30;33(2):204–14. doi: 10.1016/j.molcel.2008.12.012 (PMC2642989; doi:10.1016/j.molcel.2008.12.012)
Supplement: Document S1. Supplemental Experimental Procedures, Results, Supplemental References, and 12 Figures [file mmc1.pdf]

Molecular Cell, Volume *33*

Supplemental Data

Enhancement of the Seed-Target Recognition Step

in RNA Silencing by a PIWI/MID Domain Protein

James S. Parker, Eneida A. Parizotto, Muhan Wang, S. Mark Roe, and David Barford

AfPiwi – duplex DNA crystal structure

Nucleotides G2 to G6 adopt a B-form DNA conformation allowing their phosphate groups to form more intimate contacts to the protein compared with the equivalent nucleotides of an A-form RNA duplex. This difference in conformation allows the G4 phosphate to form a more favourable, shorter hydrogen bond with the guanidinium group of Arg380, a hydrogen bond to the amide side chain of Asn378 (not present in the AfPiwi-RNA complex) and G5 accepts a direct hydrogen bond from Arg385, possibly accounting for the higher affinity of AfPiwi for DNA (Ma et al., 2005). G6 forms no contacts to AfPiwi, whereas G7-G16 are disordered. In contrast to the exposed position of the T1 nucleotide of an RNA duplex in complex with AfPiwi, the equivalent nucleotide of the DNA duplex is inserted within a pocket of domain-A (Parker et al., 2004), interacting via van der Waals contacts (involving Ile30 and Phe151) and a hydrogen bond between the N6 amino group of the adenine base and the carboxylate of Asp154. Also unlike the RNA complex, the 3' dinucleotide overhang is disordered.

Size exclusion assay for binding enhancement

Experimental Procedures. Complexes were assembled from purified components in the ITC buffer and incubated at room temperature for at least five minutes before loading. Analysis was performed at 22°C on a Superdex-75 10/300 GL column (GE Healthcare) in the ITC buffer, running at 0.5 ml/min. 0.1 ml of sample was applied per run. The guide and target strands used were G_{12} and T_{scan1} as shown in Figure 4. Quantitation of complex formation at the various target strand concentrations (Figure S4F) was performed as follows: 1) The areas under the free target strand peaks (Figure S4D) were integrated following baseline and peak border correction with the Unicorn software (GE Healthcare) 2) The corresponding free target strand concentrations were determined from a reference run of known concentration, and 3) Complex concentrations were deduced as total minus free target strand concentrations.

Results. Size exclusion chromatography was used to separate and quantify the various components of binding reactions (complex and individual components). A preliminary run of guide and target strands in isolation and in complex (Figure S4A) showed that target strand in isolation (free target strand) was the only clearly resolved constituent. Therefore, the free target strand peak was used as the measure of the extent of complex formation. A proof-of-principle experiment (Figure S4B) showed that weakening the guide - target interaction (in isolation) via a mismatch (T:G at position 3) led to an increase in the amount of free target strand (increase in the size of the free target strand peak) at three different target strand concentrations (2, 4 and 6 μM, with guide fixed at 5 μM). Figures S4C and S4D show guide – target binding reactions at various target concentrations between 2 and 10 μM (different colours) in isolation (solid curves) and in the presence of AfPiwi (dotted curves). The data clearly show depleted free target strand

in the presence of AfPiwi at every target concentration tested (Figure S4D, compare solid and dotted curves). This is consistent with increased complex formation in each case and hence tighter binding. Quantitation of the data in Figure S4F illustrates increased complex formation in the presence of AfPiwi, with binding curves that display close similarity to theoretical curves based on the affinities obtained from ITC (Figure 4). Figure S4E illustrates that free target strand is not depleted by the addition of excess AfPiwi, and hence the depletion is not caused by direct interaction with AfPiwi.

References

Ma, J. B., Yuan, Y. R., Meister, G., Pei, Y., Tuschl, T., and Patel, D. J. (2005). Structural basis for 5'-end-specific recognition of guide RNA by the A. fulgidus Piwi protein. Nature *434*, 666-670.

Parker, J. S., Roe, S. M., and Barford, D. (2004). Crystal structure of a PIWI protein suggests mechanisms for siRNA recognition and slicer activity. Embo J *23*, 4727-4737.

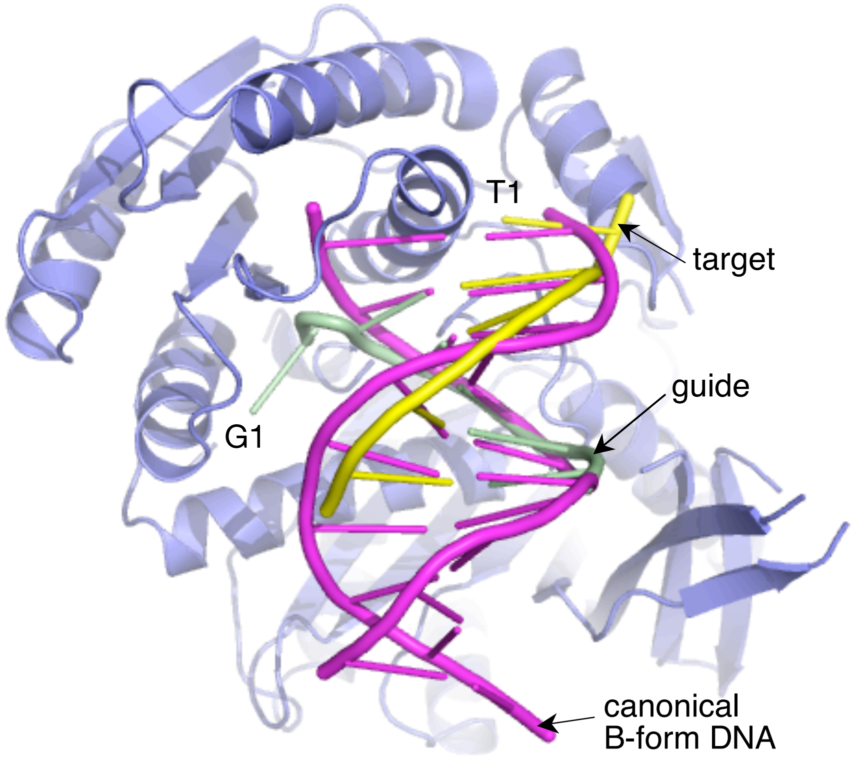

Figure S1. Superposition of the AfPiwi-DNA duplex crystal structure with canonical B-form duplex DNA (pink). Base pairs 2 – 6 of the crystallised duplex adopt B-form conformation.

```
3'- CAUCCAA-5'   RNA          3'- CAAACA-5'  RNA
    |||||||                       ||||||
5'P-TGTAGGTT-3'  DNA          5'P-UGUUUGU-3'  RNA
    12345678                      1234567
```

Interaction in isolation

Interaction with AfPiwi

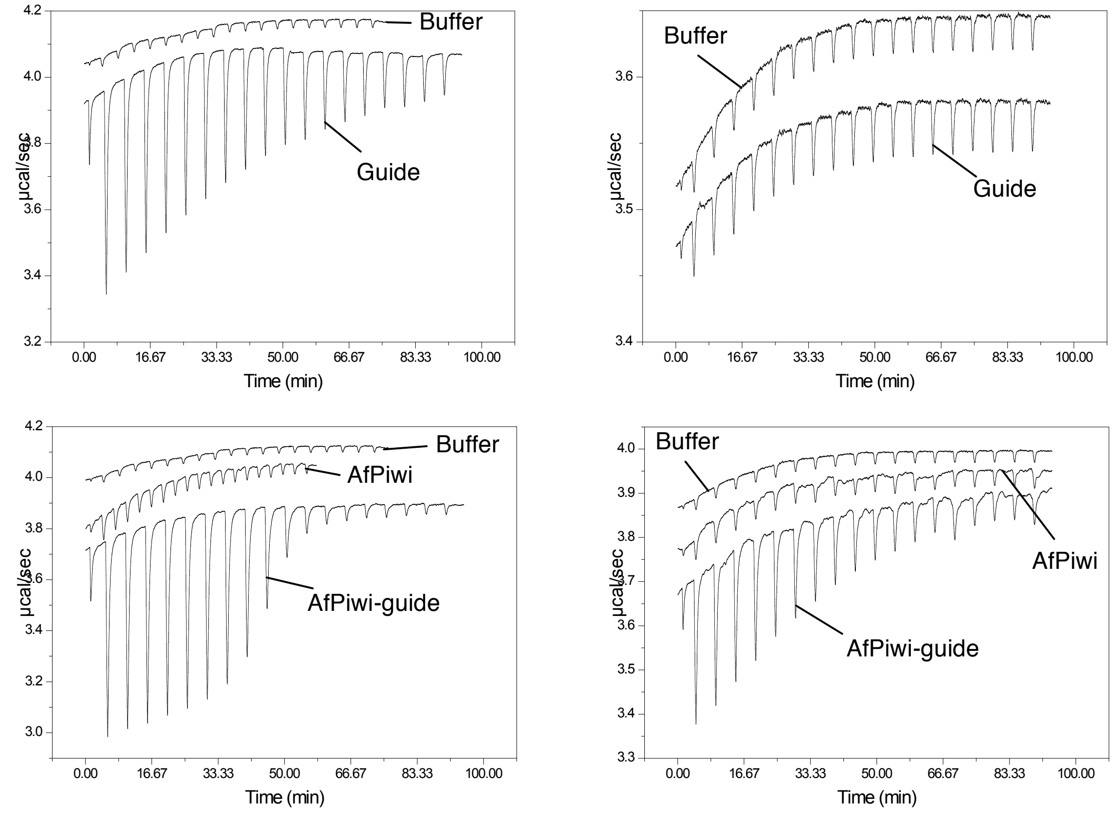

Figure S2. Raw ITC injection data for the binding experiments shown in Figure 3C (left panels) and Figure 3E (right panels). The top panels show data for the interactions in isolation and the bottom panels data for interactions in the presence of AfPiwi. Duplex structures and sequences are shown over the corresponding panels. The constituents of the ITC cell during a particular run are indicated within the panels (buffer, guide strand alone, AfPiwi alone, AfPiwi-guide complex). Raw data for the experiments in Figures 3A and 3B are shown in Figure 2B.

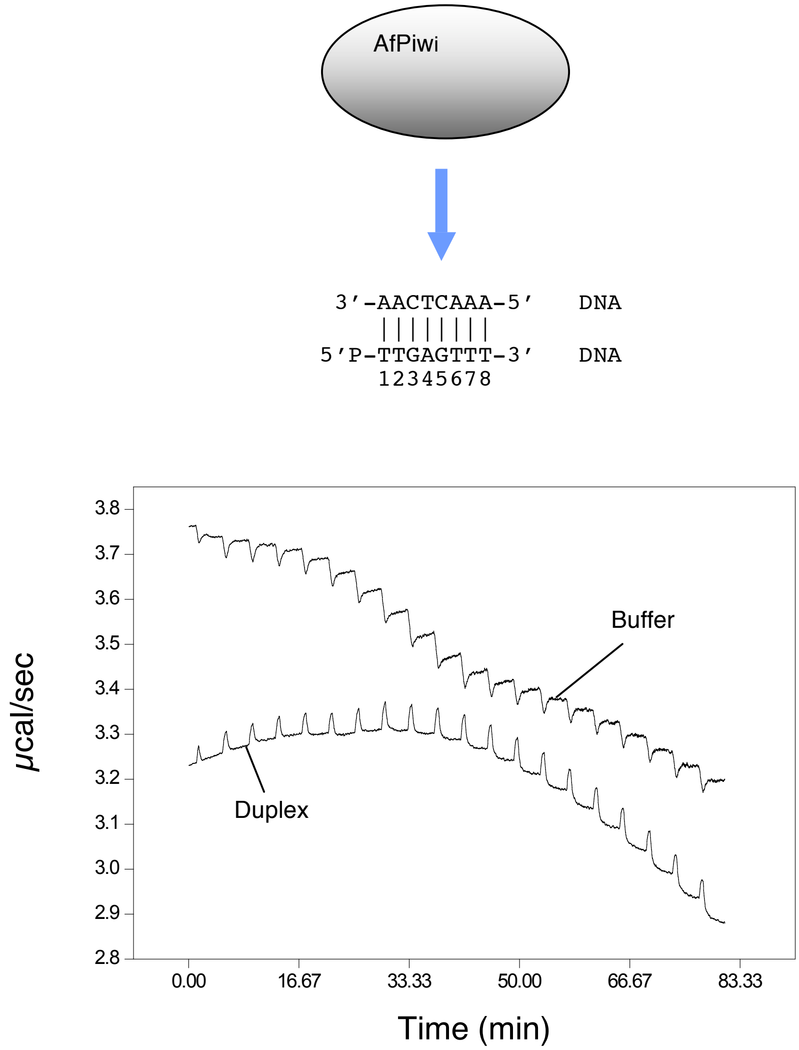

Figure S3. Interaction of AfPiwi with duplex DNA. Top, schematic of binding experiment. Duplex was in the cell to avoid the dissociation that would occur if injected from the syringe. Bottom, raw ITC injection data. Titration runs into buffer alone and duplex are indicated. AfPiwi concentration (syringe) was 18 μM, and duplex concentration (cell) was 60 μM (23 x the strand association K_d). Thus, the duplex remains under-saturated with AfPiwi throughout the experiment.

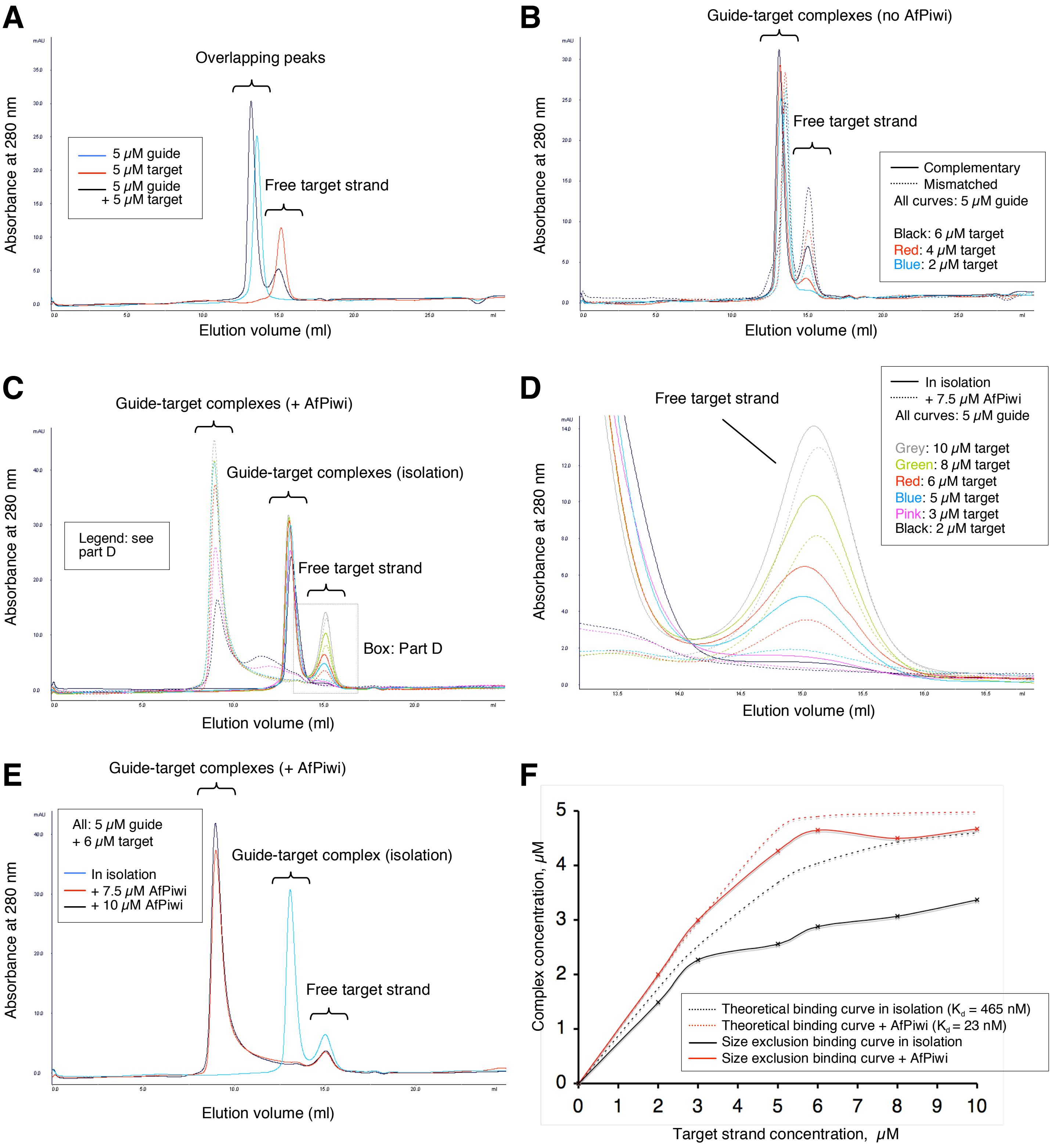

Figure S4. Size exclusion assay for binding enhancement. See Supplemental Data (supporting text) for methods and explanations. The guide and target strands used were G_{12} and T_{scan1} shown in Figure 4. (**A**) Free target strand resolves from free guide strand and guide-target duplex. (**B**) Proof-of-principle: *Weakening* the guide-target interface via a mismatch (T:G at position 3) *increases* the amount of free target strand. (**C**) Free target strand *depletion* in the presence of AfPiwi. See part D. (**D**) Zoomed in view of the 'Free target strand' peaks from part C. (**E**) Increasing the concentration of AfPiwi does not deplete free target strand. (**F**) Quantitation of data from parts C and D (solid lines), and comparison with theoretical binding curves using ITC affinities (Figure 4) (dotted lines).

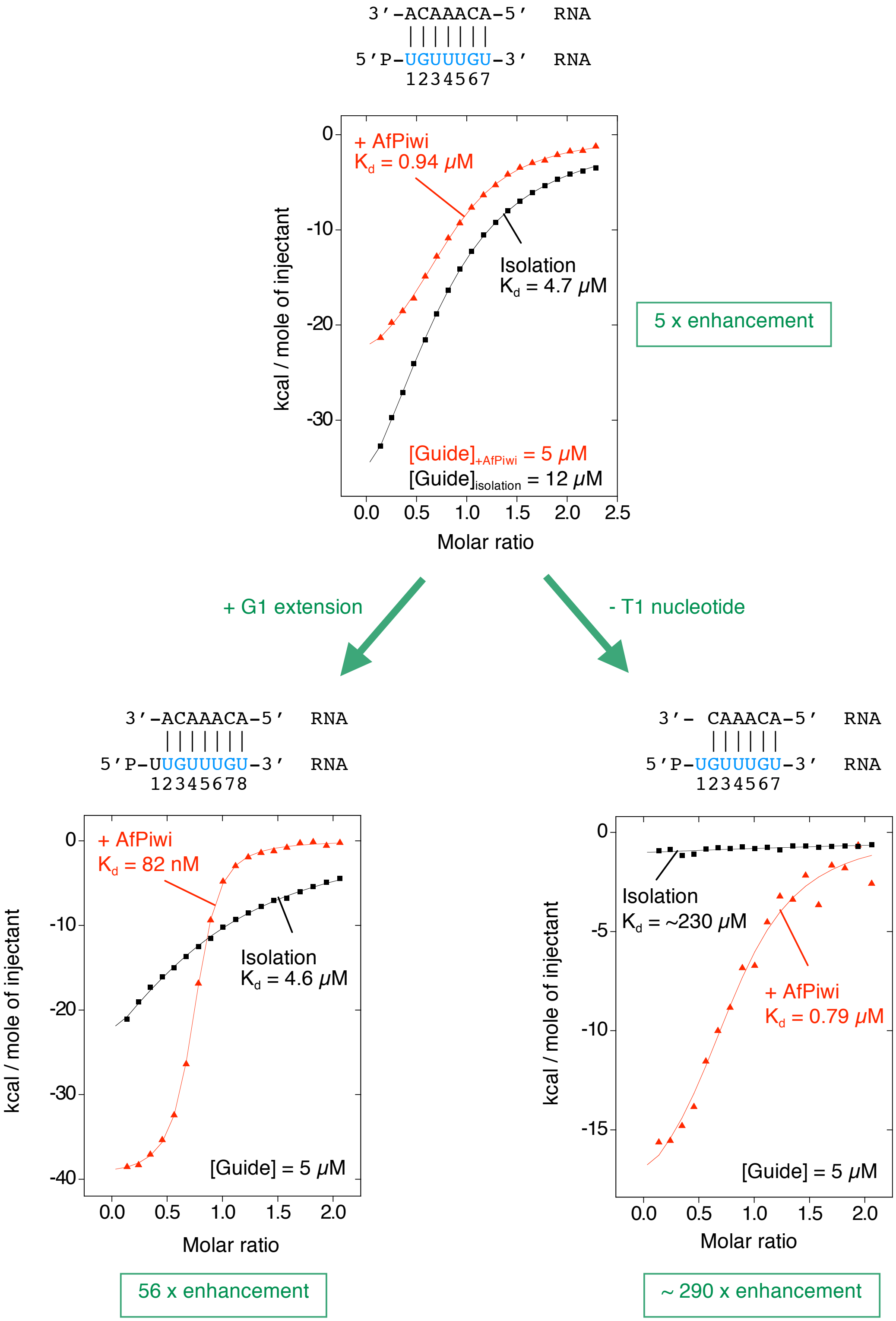

Figure S5. A flush 5' end results in a diminished *measured* enhancement for an RNA - RNA duplex. Binding data for three interactions in isolation and in the presence of AfPiwi are shown. Note that the data in the two panels at the bottom are identical to those shown in Figures 3B and 3E. (Top) The diminished *measured* binding enhancement results from loss of the G1 - T1 base pair in the presence of AfPiwi, which can be circumvented by adding an extra residue to the 5' end of the guide (bottom left) or deleting the T1 residue from the target (bottom right), so that the same number of base pairs is maintained in isolation and in the presence of AfPiwi. This effect does not occur with a DNA - DNA duplex, where the loss of the G1 - T1 base pair is compensated by the insertion of the T1 nucleotide into the T1 binding pocket in AfPiwi.

A

```
  3'-CATCCAA-5'    DNA
     |||||||
5'P-TGTAGGTT-3'    DNA
    12345678
```

B

Interaction in isolation

Interaction with AfPiwi

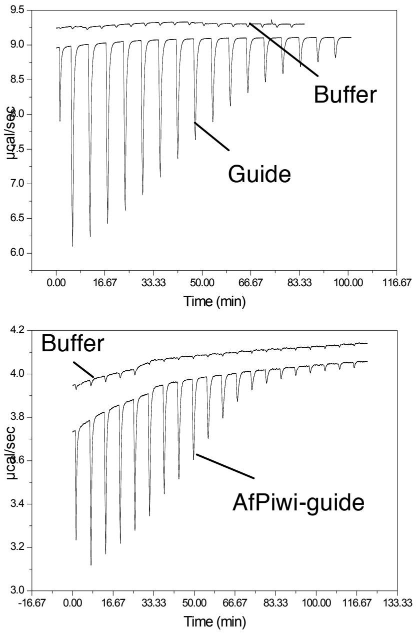

C

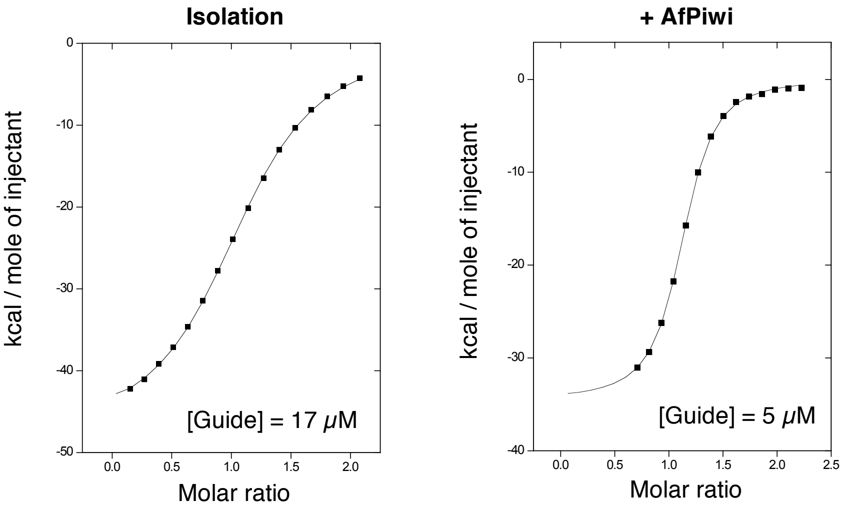

D

Interaction	N (stoichiometry)	K_d (nM)	ΔH ($kcal.mol^{-1}$)	ΔS ($cal.mol^{-1}K^{-1}$)	C ([guide]/K_d)
Isolation	1.11 ± 0.0029	2120 ± 38	-47.7 ± 0.18	-137	8.0
+ AfPiwi	1.10 ± 0.0029	99.0 ± 3.2	-34.5 ± 0.18	-85.6	51

Figure S6. Enhancement of a DNA seed - target interaction in the absence of a T1 nucleotide. (**A**) Duplex sequence and structure. (**B**) Raw ITC injection data for the interaction in isolation (top) and in the presence of AfPiwi (bottom). Titration runs into buffer alone or guide / AfPiwi-guide complex are indicated. (**C**) Corrected integrated heats and best-fit theoretical binding isotherms for the guide - target interactions in isolation (left) and in the presence of AfPiwi (right). (**D**) Thermodynamic parameters for binding reactions.

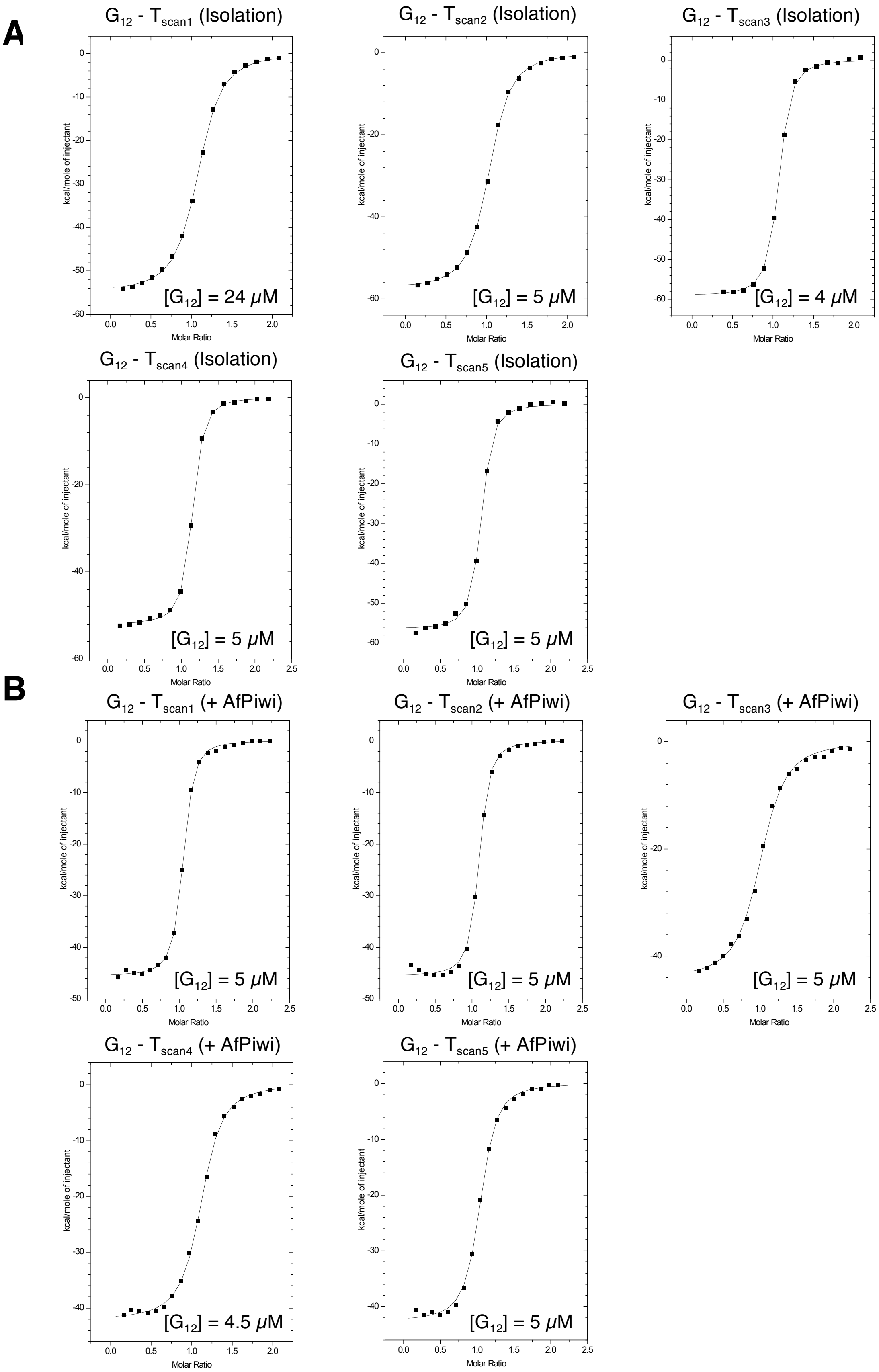

Figure S7. Corrected integrated heats and best-fit theoretical binding isotherms for the scanning experiments shown in Figure 4. (**A**) Interactions in isolation. (**B**) Interactions in the presence of AfPiwi. Raw titration data are shown in Figure S8.

A

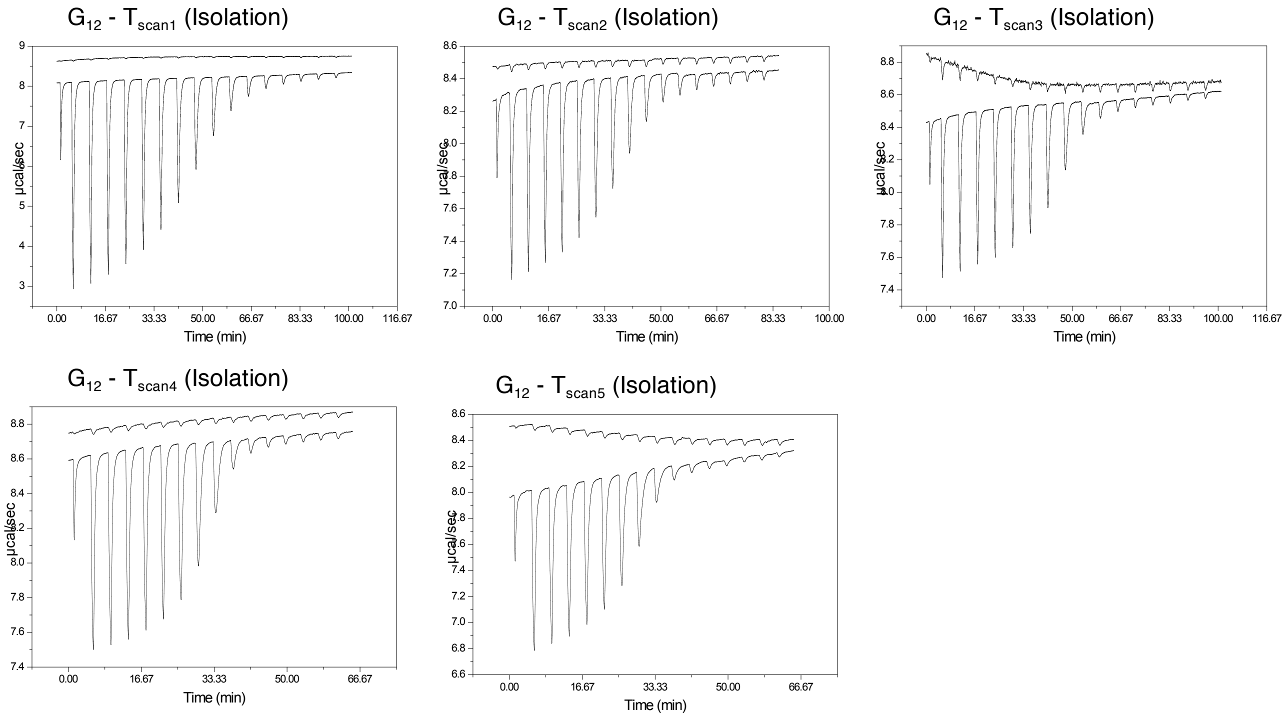

B

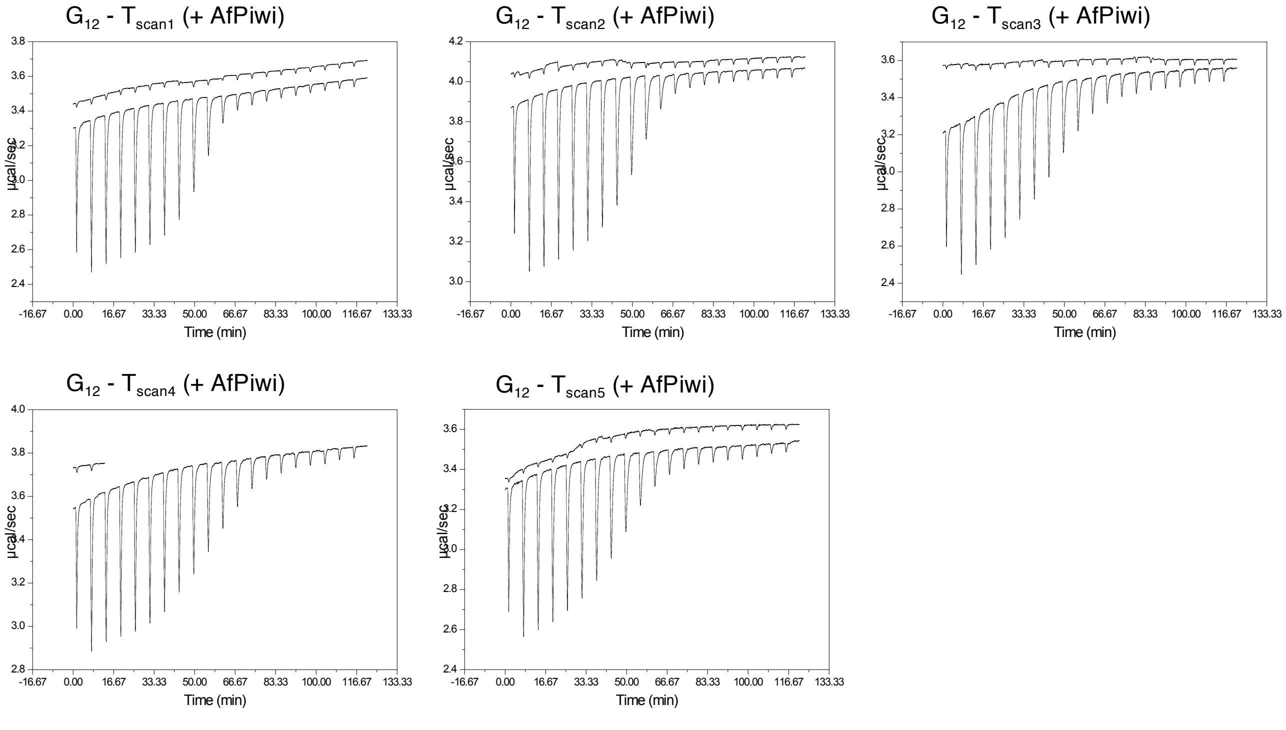

Figure S8. Raw ITC injection data for the scanning experiments shown in Figure 4. (**A**) Interactions in isolation. (**B**) Interactions in the presence of AfPiwi. In all charts, the top run is target into buffer, and the bottom run is the experimental run. Corresponding binding isotherms are shown in Figure S7.

A

```
3'-ACATCCAACGT-5'     T11 (DNA)
    |||||||||||
5'P-TGTAGGTTGCAT-3'   G12 (DNA)
    123456789012
```

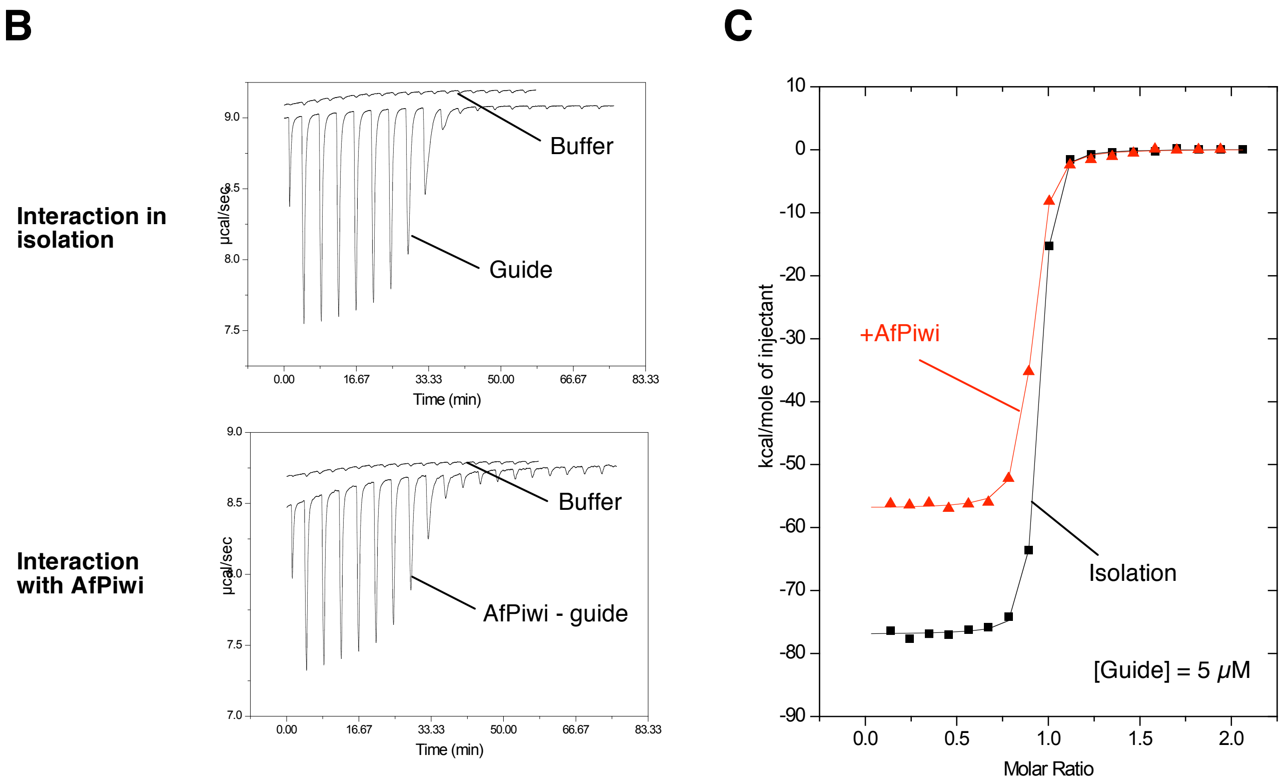

Figure S9. Raw ITC injection data and fitted binding isotherms for the modulation of the G_{12} - T_{11} interaction shown in Figure 4. (**A**) Duplex sequence and structure. (**B**) Raw ITC injection data for the interaction in isolation (top) and in the presence of AfPiwi (bottom). Titration runs into buffer alone or guide / AfPiwi-guide complex are indicated. (**C**) Corrected integrated heats and best-fit theoretical binding isotherms for the guide - target interactions in isolation (black) and in the presence of AfPiwi (red).

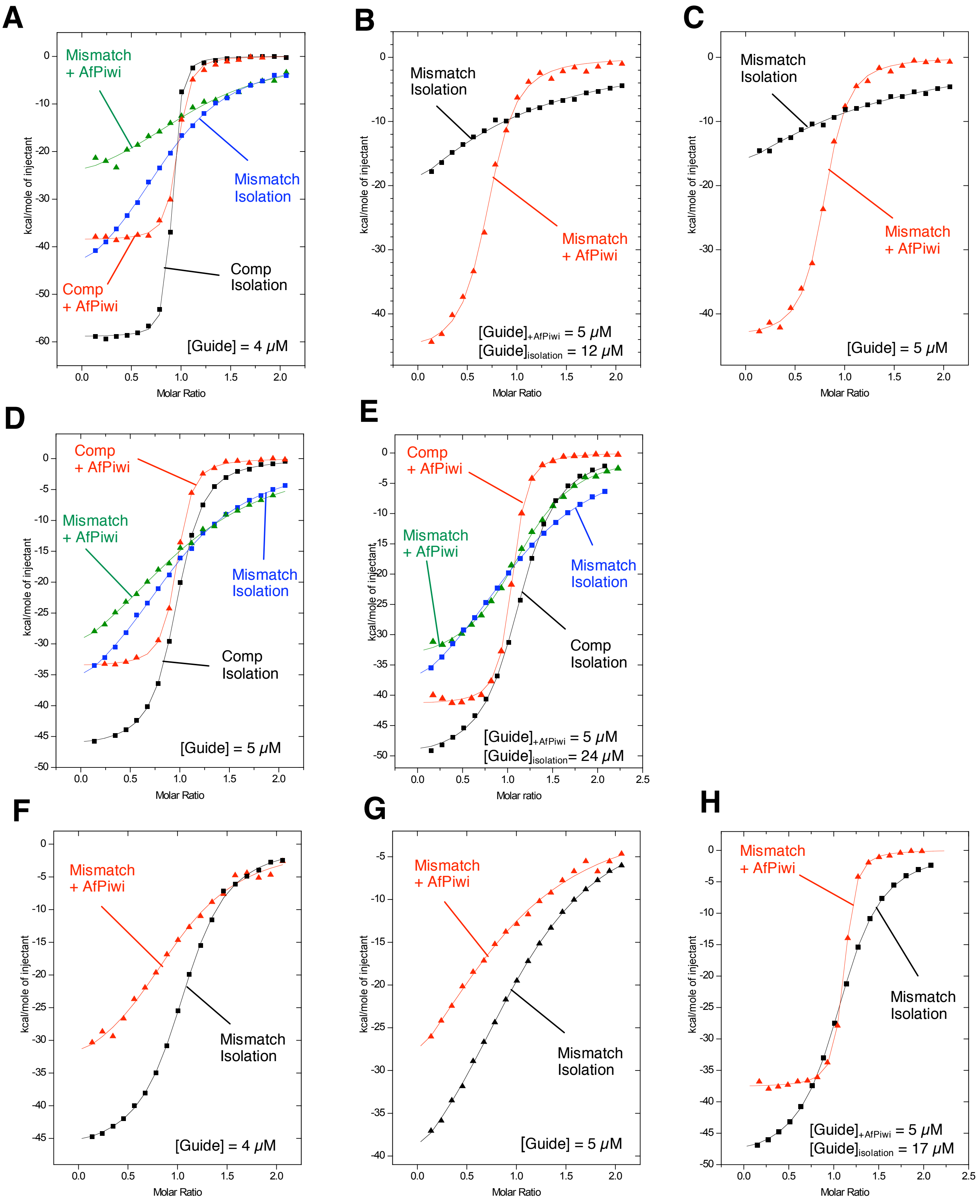

Figure S10. Corrected integrated heats and best-fit theoretical binding isotherms for the mismatch experiments shown in Figure 5. Parts **A - H** correspond to parts A - H in Figure 5, respectively. Complementary or mismatched interactions in isolation and in the presence of AfPiwi are indicated. Note that the complementary interactions for parts B, C, F, G and H are shown elsewhere in the manuscript.

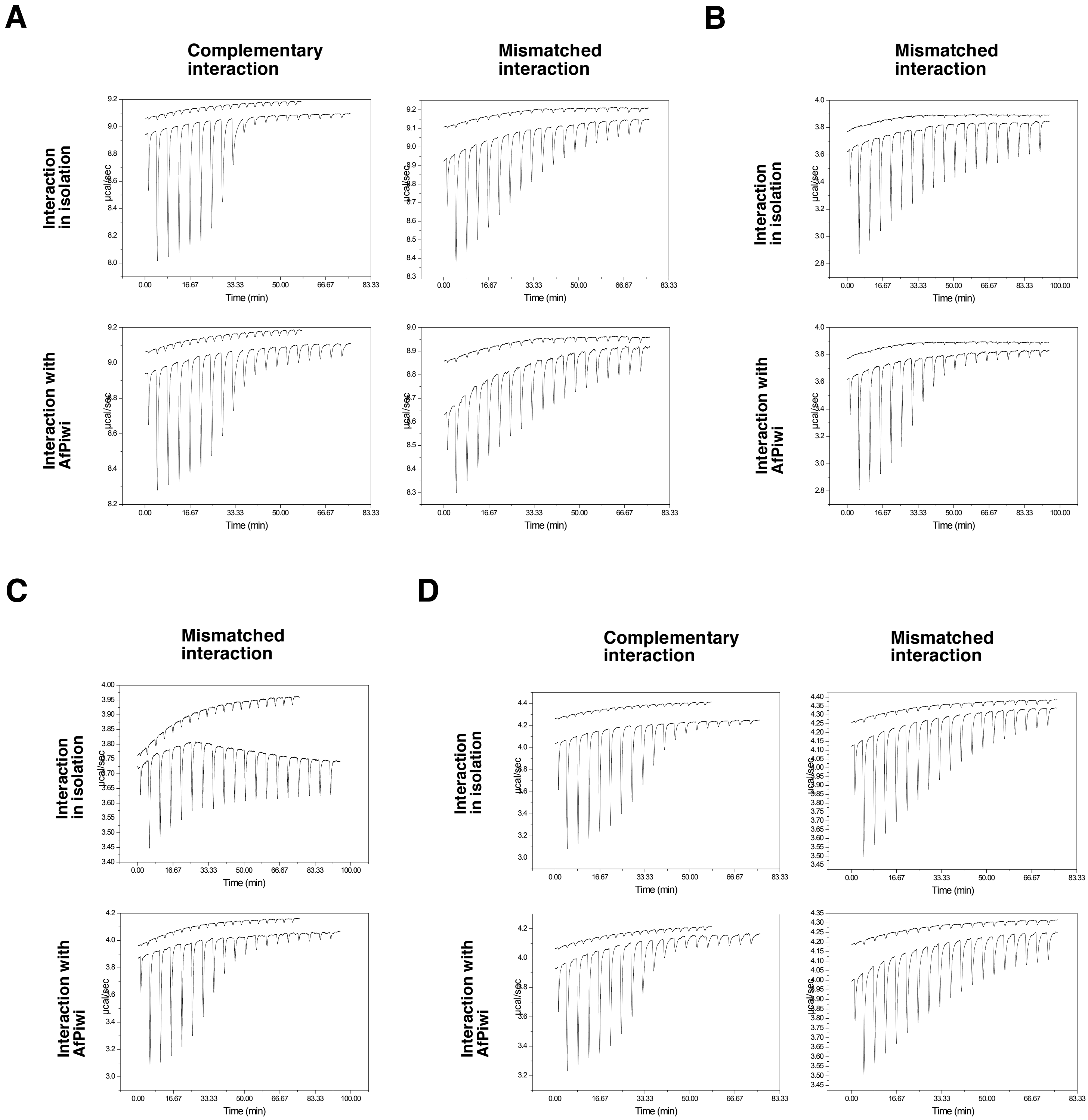

Figure S11. Raw ITC injection data for the mismatch experiments shown in Figure 5 (A - D). Parts **A** - **D** correspond to parts A - D in Figure 5, respectively. Complementary and mismatched interactions, in isolation and in the presence of AfPiwi, are indicated. In all charts, the top run is target into buffer, and the bottom run is the experimental run. Corresponding binding isotherms are shown in Figure S10. Note that the data for the complementary interactions in parts B and C are shown elsewhere in the manuscript.

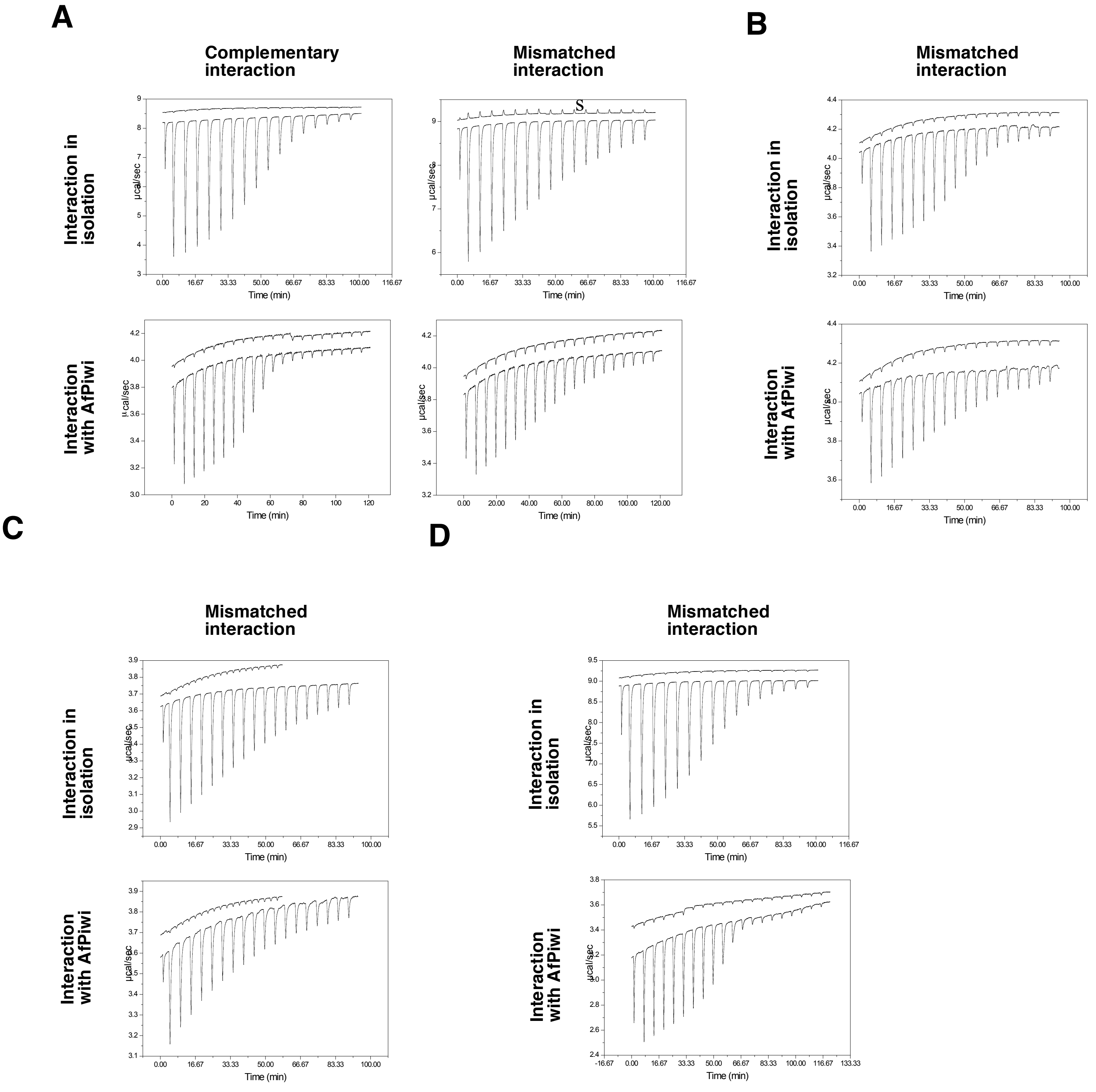

Figure S12. Raw ITC injection data for the mismatch experiments shown in Figure 5 (E - H). Parts **A** - **D** correspond to parts E - H in Figure 5, respectively. Complementary and mismatched interactions, in isolation and in the presence of AfPiwi, are indicated. In all charts, the top run is target into buffer, and the bottom run is the experimental run. Corresponding binding isotherms are shown in Figure S10. Note that the data for the complementary interactions in parts B - D are shown elsewhere in the manuscript.
